# Role of Endogenous Sulfur Dioxide in Regulating Vascular Structural Remodeling in Hypertension

**DOI:** 10.1155/2016/4529060

**Published:** 2016-09-18

**Authors:** Jia Liu, Yaqian Huang, Selena Chen, Chaoshu Tang, Hongfang Jin, Junbao Du

**Affiliations:** ^1^Department of Pediatrics, Peking University First Hospital, Beijing 100034, China; ^2^University of California, San Diego, La Jolla, CA 92093, USA; ^3^Department of Physiology and Pathophysiology, Peking University Health Science Center, Beijing 100191, China; ^4^Key Lab of Molecular Cardiovascular Science of Ministry of Education, Beijing 100191, China

## Abstract

Sulfur dioxide (SO_2_), an emerging gasotransmitter, was discovered to be endogenously generated in the cardiovascular system. Recently, the physiological effects of endogenous SO_2_ were confirmed. Vascular structural remodeling (VSR), an important pathological change in many cardiovascular diseases, plays a crucial role in the pathogenesis of the diseases. Here, the authors reviewed the research progress of endogenous SO_2_ in regulating VSR by searching the relevant data from PubMed and Medline. In spontaneously hypertensive rats (SHRs) and pulmonary hypertensive rats, SO_2_/aspartate aminotransferase (AAT) pathway was significantly altered. SO_2_ inhibited vascular smooth muscle cell (VSMC) proliferation, promoted apoptosis, inhibited the synthesis of extracellular collagen but promoted its degradation, and enhanced antioxidative capacity, thereby playing a significant role in attenuating VSR. However, the detailed mechanisms needed to be further explored. Further studies in this field would be important for the better understanding of the pathogenesis of systemic hypertension and pulmonary hypertension. Also, clinical trials are needed to demonstrate if SO_2_ would be a potential therapeutic target in cardiovascular diseases.

## 1. Introduction

Since the 1980s, studies have shown that the endogenous gaseous molecules nitric oxide (NO), carbon monoxide (CO), and hydrogen sulfide (H_2_S) are endogenously produced and have a wide range of biological effects including vasodilation and inhibition of vascular smooth muscle cell (VSMC) proliferation and platelet aggregation. They have an important physiological and pathological significance. Gaseous signal molecules share the following characteristics [1]: (1) small gaseous molecules with low molecular weight; (2) freely passing through the cell membrane, independent of the specific cell receptor; (3) being endogenously generated under the enzyme catalysis and regulated by metabolic pathways; (4) having a clear specific function at physiological concentration; and (5) exerting biological effects, having specific cellular and molecular targets. Accordingly, gaseous signal molecule pathways and the physiologic and pathophysiologic significance have become hot topic in cardiovascular system and other systems [2].

In recent years, studies showed endogenous SO_2_ pathways in tissues of rats including cardiovascular tissues [3, 4]. The physiological effects of SO_2_ were also confirmed, including vasodilation [4–6], negative regulation of cardiac function [7], and regulation of lipid metabolism [8–10]. Also, the pathophysiologic role of SO_2_ in the processes of vascular structural remodeling (VSR) [11, 12], inflammatory response [13], and oxidative response was indicated [12, 13]. In this review, the research progress of SO_2_ in regulating VSR was summarized.

## 2. The Property of SO_2_

### 2.1. General Physical and Chemical Properties of SO_2_

SO_2_ is a colorless gas accompanied by a pungent odor [14]. The relative molecular mass of SO_2_ is 64 g/mol, and the ionic charge of sulfur is 4+. Hence, it has both oxidative ability and reducibility. Reduced sulfur has a longer half-life in the body, and thereby it has a strong antioxidant effect [15]. In the atmosphere, however, SO_2_ is oxidized to sulfuric acid or sulfate aerosols which severely pollute the atmosphere [16].

### 2.2. Generation and Metabolism of Endogenous SO_2_

**Figure 1:**
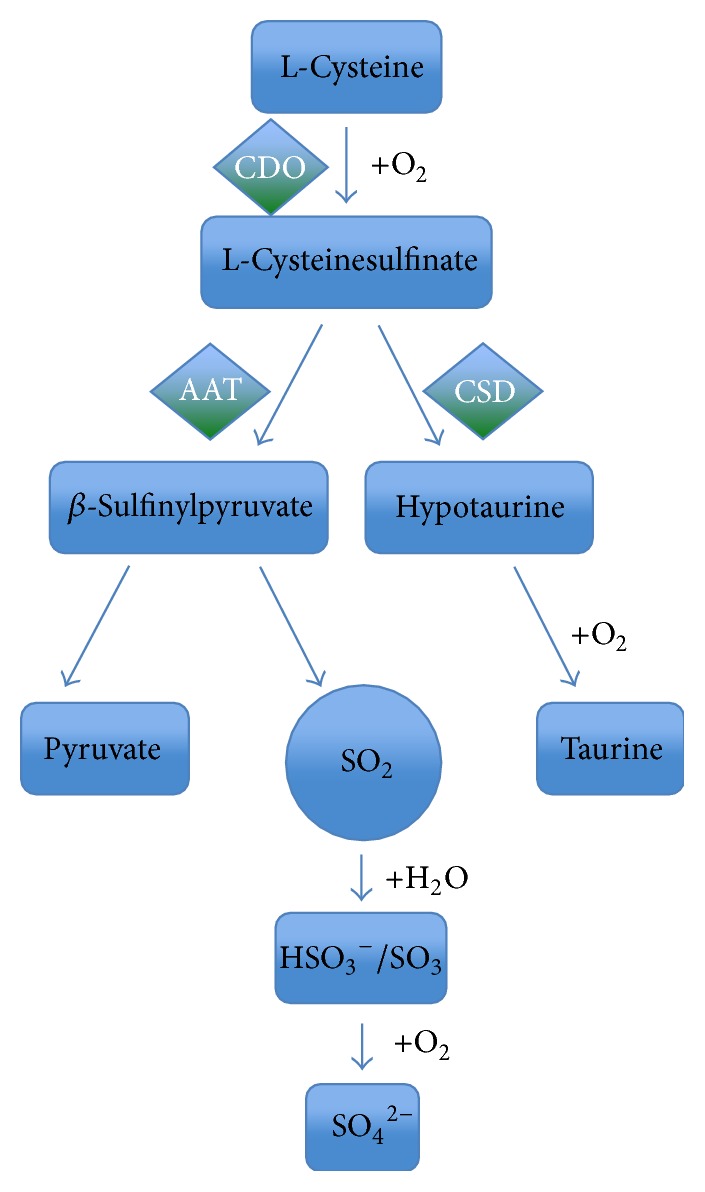
Endogenous generation of sulfur dioxide. Firstly, a sulfur-containing amino acid is metabolized to L-cysteine that is then oxidated into L-cysteinesulfinate under the role of CDO. L-Cysteinesulfinate, an analogue of L-asparaginic acid, can be transaminated into *β*-sulfinylpyruvate under the role of AAT, which is spontaneously decomposed into pyruvate and SO_2_.* In vivo*, SO_2_ can produce HSO_3_^−^/SO_3_^2−^ (molar ratio of 1 : 3) after combining with water, which can be oxidated into SO_4_^2−^ by sulfite oxidase and then excreted through the kidneys. On the other hand, L-cysteinesulfinate can be also decarboxylated into CO_2_ and hypotaurine under the role of CSD. A large majority of hypotaurine can be further oxidized into taurine. SO_2_: sulfur dioxide; CDO: cysteine dioxygenase; CSD: cysteinesulfinate decarboxylase; AAT: aspartate aminotransferase.

Endogenous SO_2_ is produced during metabolism of sulfur-containing amino acids (Figure 1). Firstly, sulfur-containing amino acid is metabolized to L-cysteine that is then oxidized into L-cysteinesulfinate catalyzed by cysteine dioxygenase (CDO). L-Cysteinesulfinate, an analogue of L-asparaginic acid, can be transaminated into *β*-sulfinylpyruvate catalyzed by aspartate aminotransferase (AAT), which is then spontaneously decomposed into pyruvate and SO_2_ [15, 17, 18].* In vivo*, SO_2_ produces HSO_3_^−^/SO_3_^2−^ (molar ratio of 1 : 3) in water, which can be oxidated into SO_4_^2−^ by sulfite oxidase and then excreted through the kidneys [19]. However, there are still some gaseous forms of SO_2_ existing in the body. On the other hand, L-cysteinesulfinate can be also decarboxylated into CO_2_ and hypotaurine by cysteinesulfinate decarboxylase (CSD). A large majority of hypotaurine can be further oxidized into taurine, which takes place during the metabolism of bile acids. Studies have found that taurine functions as an inhibitory neurotransmitter, membrane stabilizing factor, and broad spectrum cytoprotector, and so forth [20, 21].

In addition, endogenous SO_2_ can be generated by intracellular H_2_S. Firstly, H_2_S is oxidized to thiosulfate by heme compounds, metal-protein complexes, and ferritin. Thiosulfate reacts with reduced glutathione under the catalysis of thiosulfate reductase (TSR) to form sulfite or SO_2_ [19, 22]. Meanwhile, Mitsuhashi et al. [23] found that activated neutrophils in mammals could be catalyzed by NADPH to convert H_2_S into sulfites through oxidative stress.

### 2.3. Detection Methods of SO_2_

In 2003, Balazy et al. [24] detected SO_2_ in the coronary artery and myocardium using gas chromatography/mass spectrometry. In 2008, Du et al. [4] first found that there was an endogenous SO_2_/AAT pathway in cardiovascular system and other systems, with the highest SO_2_ content in the arteries. In arteries, the highest SO_2_ content was found in the aorta (5.55 ± 0.35 *μ*mol/g protein), followed by the pulmonary artery (3.27 ± 0.21 *μ*mol/g protein), mesenteric artery (2.67 ± 0.17 *μ*mol/g protein), renal artery (2.50 ± 0.20 *μ*mol/g protein), and the caudal artery (2.23 ± 0.19 *μ*mol/g protein). The content in heart (1.78 ± 0.12 *μ*mol/g protein), liver (1.74 ± 0.16 *μ*mol/g protein), lung (1.42 ± 0.11 *μ*mol/g protein), and kidney (0.95 ± 0.11 *μ*mol/g protein) was relatively low. The content of SO_2_ in plasma was 15.54 ± 1.68 *μ*mol/L.

AAT, a key enzyme in the generation of endogenous SO_2_, is distributed in mammals [25]. There are two kinds of isoenzymes AAT1 and AAT2. AAT1 is mainly located in the cytoplasm, and AAT2 is mainly in mitochondria [26]. However, the activity of AAT in tissues was not consistent with SO_2_ tissue level. AAT activity was highest in heart tissue (4469 ± 278 U/g), followed by liver (1328 ± 198 U/g), kidney (381 ± 48 U/g), and lung tissue (175 ± 38 U/g). AAT activity value in the arteries was (188 ± 30 U/g) in renal artery, (143 ± 36 U/g) in caudal artery, (112 ± 15 U/g) in mesenteric artery, (96 ± 12 U/g) in pulmonary artery, and (88 ± 11 U/g) in the aorta. Serum AAT activity was (87 ± 18 U/g). The distribution of AAT mRNA levels was consistent with AAT activity in rats. In heart, liver, kidney, and other tissues, AAT mRNA levels were significantly higher than those found in the artery [3].

The synthesis of SO_2_ is regulated by a variety of factors. Balazy et al. [24] showed that acetylcholine (Ach) could cause an increased synthesis of SO_2_ in porcine coronary artery. However, Meng et al. [27] found that endogenous Ach could promote the generation of SO_2_ in rat vascular tissue in cultured vascular endothelial and VSMCs in a dose-dependent manner, but noradrenaline (NE) suppressed the generation of SO_2_. Factors regulating SO_2_ generation need further studies.

## 3. Vascular Structural Remodeling

In 1989, Baumbach and Heistad [28] first proposed the concept of “vascular structural remodeling” when studying the changes in cerebral artery of rats with chronic hypertension. With the wide use of pathological morphology, microstructure morphologic metrology, vascular perfusion* in vitro,* and other technologies, the study on VSR has been deepened. VSR is defined as any structural changes of blood vessels [29]. The most typical characteristics of VSR include intimal endothelial cell swelling, medial SMC hyperplasia, and adventitia extracellular matrix (ECM) deposition [29]. Gibbons and Dzau [29] pointed out that VSR was a dynamic process; it relied on the interaction among haemodynamics, other mechanical stimuli with locally produced growth factors, including signal perception, signal transduction, and synthesis and release of regulatory factors. This eventually leads to changes in the structure of the vessel wall, simultaneously accompanied by decreased vascular wall compliance, changes in the release of vasoactive substances, and other functional disorders.

## 4. SO_2_ and Vascular Remodeling in Hypertension

### 4.1. The Role of SO_2_ in the Development of Vascular Structural Remodeling in Systemic Hypertension

VSR is one of the important issues in pathogenesis of hypertension [30]. The characteristics of VSR in the process of hypertension include the hyperplastic medial SMCs, the swollen endothelial cells, and the increased ECM [31, 32]. Dzau et al. [33] summarized it up to four types: (1) both the media and intima are thickened; the inner diameter is shortened, resulting in an increased ratio of vessel wall thickness to inner diameter. This type of change is mainly due to the hypertrophy and proliferation of VSMCs. Or the proliferation may not be obvious; however, rearrangement of VSMCs and noncellular components may be significant; (2) both the inner and outer diameter are increased, suggesting that the hypertrophy of vessel wall is relatively mild. In this case, the ratio of vessel wall thickness to inner diameter is decreased. This change is due to the rearrangement of VSMCs and the proliferation is not obvious; (3) both the inner and outer diameter are decreased; and (4) the number of microcirculations is decreased.

SO_2_ plays an important role in the development of VSR in hypertension. In spontaneously hypertensive rats (SHRs), Zhao et al. [34] found that SO_2_ content and AAT activity in plasma and thoracic aorta were significantly decreased, but the ratio of vessel wall thickness and inner diameter was significantly increased, along with the accumulation of collagen I and collagen III in the aorta. However, intraperitoneal injection of SO_2_ derivatives (Na_2_SO_3_/NaHSO_3_, 0.54 mmol/kg: 0.18 mmol/kg, for five weeks) could significantly reduce the VSR and lower the blood pressure of SHRs. The results indicated that the downregulated endogenous SO_2_ pathway was involved in the VSR in hypertension.

Excessive proliferation of VSMCs is one of the key mechanisms involved in hypertensive VSR. Under the hypertensive condition, VSMCs switch from a contractile to a synthetic phenotype, with a high rate of proliferation. The abnormal proliferation of VSMCs leads to a variety of cytokines secretion and a marked ECM production, which finally results in a decrease of lumen ratio and an increase of media-lumen ratio [35]. Zhao et al. [34] found that, in SHRs, aortic tunica media thickness and VSMCs proliferation index were significantly decreased after the exogenous supplement of SO_2_ derivatives (0.54 mmol/kg: 0.18 mmol/kg, for five weeks). Liu et al. [36] found that SO_2_ inhibited vascular proliferation by suppressing the progression of the cell cycle from G1 to S phase. Going further, AAT1 and AAT2 overexpression inhibited serum-induced VSMCs proliferation of rats, whereas AAT1 and AAT2 knockdown showed an opposite effect. These results showed that endogenous SO_2_ had a negative effect on VSMCs proliferation. For the purpose of further exploring the potential mechanisms, a cell proliferation model stimulated by platelet derived growth factor-BB (PDGF-BB) was established. It was found that SO_2_ dephosphorylated the active sites of ERK1/2, MAPK kinase 1/2, and c-Raf. Furthermore, SO_2_ increased the AC activity of VSMCs of rats, thereby increasing the intracellular cAMP levels and activating PKA signaling molecules, which resulted in an increase in the Ser259 phosphorylation, an inhibitory site of Raf-1 molecule, thereby inhibiting the Raf-1 kinase activity and leading to the inactivation of the ERK/MAPK pathway [36]. The results suggested that SO_2_ could attenuate VSR through suppressing proliferation of VSMCs. And cAMP/PKA/ERK/MAPK signaling was involved in the inhibitory effect of SO_2_ on VSMC proliferation.

Numerous studies indicated that the VSMC apoptosis participated in the development of VSR. Zhao et al. [37, 38] found that, in SHRs, the exogenous supplement of SO_2_ derivatives (0.54 mmol/kg: 0.18 mmol/kg, for five weeks) could promote apoptosis of VSMCs, and its mechanism might be related to inhibited Bcl-2 and activated Fas, ultimately inducing apoptosis through the molecule caspase-3. These data suggested that SO_2_ inhibited VSR in hypertension in association with promoting VSMC apoptosis.

### 4.2. The Role of SO_2_ in the Development of Vascular Structural Remodeling in Pulmonary Artery Hypertension

Pulmonary artery hypertension (PAH) is a pathophysiologic syndrome that leads to pulmonary vascular bed obstruction and progressively increased pulmonary vascular resistance, ultimately resulting in right heart failure. It is well known that pulmonary VSR contributes to all types of pulmonary hypertension. The main pathologic characteristics of pulmonary VSR in pulmonary artery hypertension include thickening of the adventitial, medial and/or intimal layer of the pulmonary arteries, elevated stiffening of the elastic pulmonary arteries, vascular bed occlusive lesions, proliferation of pulmonary fibroblasts, SMCs and endothelial cells, and excessive accumulation of ECM [39]. Interestingly, SO_2_ plays an important role in the development of pulmonary artery hypertension, including hypoxic pulmonary hypertension, high pulmonary blood flow-induced pulmonary hypertension, and monocrotaline-induced pulmonary hypertension.

#### 4.2.1. Downregulation of Endogenous SO_2_ Pathway Is Involved in the Development of Pulmonary VSR in Hypoxic Pulmonary Hypertension

Hypoxic pulmonary vasoconstriction caused by acute hypoxia and hypoxic pulmonary vascular structural remodeling caused by chronic hypoxia are the main pathophysiological processes of hypoxic pulmonary hypertension [40]. In comparison to the response of systemic blood vessels, the response of pulmonary vasculature to hypoxia has its specific characteristics; that is, pulmonary vascular reactive constriction in hypoxic condition can help maintain arterial oxygen saturation, which has an important physiological significance. However, if hypoxia persists, pulmonary arteriole will develop structural remodeling and pulmonary artery pressure will stay at a high level. And pulmonary hypertension will lead to aggravated hypoxia, and form a vicious circle. Interestingly, SO_2_ content in the plasma and lung tissues from rats with hypoxic pulmonary hypertension was significantly decreased, as well as AAT1 mRNA expression and AAT activity [11]. In order to examine the significance of downregulated SO_2_ pathway in the development of hypoxic pulmonary hypertension, investigators administered SO_2_ derivatives to the hypoxic rats (0.54 mmol/kg: 0.18 mmol/kg, for three weeks). The results showed that it could significantly reduce mean pulmonary arterial pressure of the hypoxic rat [11]. Meanwhile, SO_2_ derivatives significantly alleviated hypoxic pulmonary VSR, as demonstrated by a reduced percentage of muscular arteries and an increased percentage of nonmuscular arteries. Moreover, the relative medial thickness and relative medial areas of muscular arteries were decreased in hypoxic rats after SO_2_ treatment. The above results implied that the reduction of endogenous SO_2_ was involved in the development of hypoxic pulmonary VSR.

The studies have shown the advances in the mechanisms by which SO_2_ plays a crucial role in the development of hypoxic pulmonary VSR. The deposition of ECM, including collagen, elastin, proteoglycans, and glycoproteins, participates in pulmonary VSR. Collagen deposition plays a crucial role in VSR. Exogenous supplementation of SO_2_ derivatives could alleviate pulmonary VSR and attenuate pulmonary hypertension. Furthermore, SO_2_ could significantly reduce the mRNA expressions of procollagens I and III in hypoxic pulmonary hypertension rats [11]. On the contrary, the mRNA levels of procollagens I and III were markedly increased in hypoxic pulmonary hypertensive rats administrated with hydroxamate (HDX), an inhibitor of AAT. The results indicated that SO_2_ inhibited hypoxic hypertension-induced pulmonary VSR possibly by the suppression of collagen deposition. It is known that reduced collagen degradation has an important significance in pulmonary hypertensive VSR. It is regulated by the balance between matrix metalloproteinase (MMP) and the tissue inhibitor of metalloproteinase (TIMP). Zaidi et al. [41] found that MMPs activity was increased in the development of hypoxic pulmonary hypertension. Vieillard-Baron et al. [42] transferred the TIMP-1 gene into the lungs of rats by adenovirus, which successfully inhibited the expression and activity of MMPs, preventing from hypoxic pulmonary hypertension and pulmonary VSR. SO_2_ was found to increase the mRNA ratio of MMP-13/TIMP-1 in pulmonary arteries of hypoxic pulmonary hypertensive rats by increasing MMP-13 mRNA level and decreasing TIMP-1 mRNA expression [11]. These data suggested that SO_2_ attenuated hypoxic pulmonary VSR at least partly by reducing collagen degradation.

Additionally, SO_2_ inhibited pulmonary VSMCs proliferation in association with the suppression of Raf-1 protein and the downstream ERK/MAPK pathway in hypoxic pulmonary hypertensive rats. Moreover, Bai and Meng [43] found that the exogenous inhalation of SO_2_ downregulated mRNA levels of Bcl-2, increased protein expressions of p53 and Bax, and enhanced caspase-3 activity in rat lung, suggesting that SO_2_ alleviated hypoxic pulmonary VSR by maintaining the balance between pulmonary vascular cell proliferation and apoptosis.

#### 4.2.2. Downregulated Endogenous SO_2_ Pathway Is Involved in the Development of Pulmonary VSR in High Pulmonary Blood Flow-Induced Pulmonary Hypertension

Pulmonary hypertension induced by high pulmonary blood flow is one of the most common complications of left-to-right shunt congenital heart disease. Increased pulmonary blood flow could result in pulmonary VSR and finally the development of pulmonary hypertension. Pronounced medial thickening and increased collagen content in pulmonary arteries were observed in cases of congenital heart disease accompanied with pulmonary hypertension [44]. Luo et al. [45] developed the rat model of high pulmonary blood flow-induced pulmonary hypertension by systemic-pulmonary shunting. They found that the content of SO_2_, the mRNA, and protein expression of AAT2 as well as AAT activity in pulmonary vessels were decreased in pulmonary hypertensive rats. The administration of SO_2_ derivatives (0.54 mmol/kg: 0.18 mmol/kg, for eight weeks) could significantly reduce the mean pulmonary artery pressure and improve the pulmonary vascular pathological changes of the rats, as demonstrated by the decreased percentage of muscularized pulmonary arteries. Furthermore, Liu et al. [46] found that SO_2_ alleviated the protein expression of collagen I and collagen III.

However, the mechanisms by which SO_2_ alleviates VSR are incompletely understood. It was suggested that endogenous SO_2_ might alleviate pulmonary VSR via upregulating the reduced endogenous H_2_S pathway [45, 47, 48]. To further investigate the signaling pathway by which SO_2_ alleviated pulmonary VSR, Liu et al. used a Flexcell Fx-5000 Tension System to establish a cell model to mimic the mechanical stretching of high blood flow on vascular wall in high blood flow induced pulmonary hypertension. They found that mechanical stretching could downregulate endogenous SO_2_/AAT1 pathway in pulmonary fibroblasts (PAFs) and then activate TGF-*β*1/Smad2/3 pathway, which ultimately resulted in an excessive collagen synthesis. Overexpression of AAT1, however, could antagonize the activation of TGF-*β*1/Smad2/3 pathway caused by mechanical stretching, which could be exaggerated by knockdown of AAT1. Furthermore, in the rat model of high pulmonary blood flow-induced pulmonary hypertension by surgical systemic-pulmonary shunting, the activated TGF-*β*1/Smad2/3 pathway could be inhibited by the administration of SO_2_ derivatives [46]. The above results suggested that in high pulmonary blood flow-induced pulmonary hypertension the downregulated endogenous SO_2_ activated TGF-*β*1/Smad2/3 pathway, which ultimately resulted in collagen remodeling.

#### 4.2.3. Upregulated Endogenous SO_2_ Pathway Is Involved in the Development of Pulmonary VSR in Monocrotaline-Induced Pulmonary Hypertension

Monocrotaline (MCT), a pyrrolizidine alkaloid, is metabolized into MCT pyrrole in the liver. The substance of MCT induces pulmonary hypertension in a rat model by causing pulmonary artery smooth muscle hypertrophy, inflammation, and endothelial cells injury. Mean pulmonary artery pressure (mPAP) and the ratio of right ventricle to left ventricle plus septum were increased markedly in the MCT-treated rats [12]. Meanwhile, pulmonary VSR developed accompanied with the increased SO_2_ level, AAT activity, and mRNA expression. SO_2_ administration significantly alleviated pulmonary VSR and reduced mPAP, while HDX aggravated them in the MCT-treated rats [12]. It suggested that endogenous SO_2_ might play a protective role in the MCT-induced pulmonary VSR in pulmonary hypertensive rats.

Studies have suggested that enhanced oxidative stress by increasing superoxide anions and other reactive oxygen species production was involved in the pathophysiology of MCT-induced pulmonary hypertensive VSR [49, 50]. Superoxide dismutase (SOD), glutathione peroxidase (GSH-Px), glutathione (GSH), and catalase (CAT) are antioxidant enzymes, and the malondialdehyde (MDA) is oxidation product. Although inhalation of SO_2_ was considered to cause oxidative damage to mammals [51], the endogenous SO_2_ at a low level was found to have an antioxidant effect in MCT-induced pulmonary hypertension. In MCT-induced pulmonary hypertensive rats [12], the activities of SOD, GSH-Px, GSH, CAT, and MDA were elevated in lung tissues in association with a protective upregulated SO_2_ level. With supplement of SO_2_ derivatives, the content of SOD, GSH-Px, and CAT went higher, while inhibition of endogenous SO_2_ with HDX suppressed the activities of SOD and CAT [12]. These data indicate that upregulated SO_2_ production plays a protective role in pulmonary VSR by promoting endogenous antioxidative capacity.

## 5. Conclusion

SO_2_ was previously recognized as an industrial waste gas. Environmental SO_2_ can cause oxidative damage to the cardiovascular system, respiratory system, and other systems [51]. SO_2_ and sulphites can cause DNA damage in mammalian cells, such as chromatin breakage, sister chromatid exchange, micronucleus formation, DNA-protein cross-linking, and other stages [52, 53]. In recent years, however, SO_2_ as discussed herein is found to have the characteristics of gaseous molecules such as endogenous continuous generation, fast transmission, extensive action, and low molecular weight and therefore play an important role in the physiology and pathophysiology of cardiovascular diseases.

**Figure 2:**
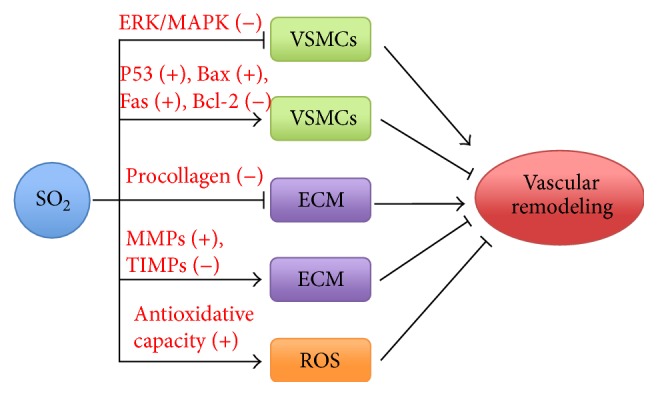
Mechanisms by which SO_2_ attenuates VSR. SO_2_ attenuates VSR by inhibiting proliferation of VSMCs, promoting apoptosis, inhibiting the synthesis of extracellular collagen, promoting the degradation of extracellular collagen, and increasing the antioxidative capacity. SO_2_: sulfur dioxide; VSMCs: vascular smooth muscle cells; ECM: extracellular matrix; ROS: reactive oxygen species.

VSR is an important pathogenic base of cardiovascular diseases, such as systemic hypertension and pulmonary hypertension. In the process of VSR in systemic hypertension or pulmonary hypertension, VSMC proliferation is excessive while apoptosis reduced, and extracellular collagen synthesis and degradation are imbalanced, leading to an excessive deposition of collagen in the vascular wall. Although the increase in collagen content may help to resist the excessive stress in the vessel wall and maintain the integrity of blood vessels, the excessive collagen deposition can reduce the compliance and increase the resistance of the vascular wall, which has become an important factor in deterioration of systemic hypertension or pulmonary hypertension. Since VSR is a core pathological change of systemic hypertension and pulmonary hypertension, it is important to further explore its mechanisms in order to better understand its pathogenesis, which would ultimately provide scientific basis for potential therapeutic targets.* In vitro *and* in vivo* experiments demonstrated that downregulated SO_2_/AAT pathway was involved in VSR of systemic hypertension and pulmonary hypertension. Furthermore, SO_2_ has been shown to play an important regulatory role in VSR by inhibiting VSMC proliferation and the synthesis of extracellular collagen and promoting VSMC apoptosis and collagen degradation. In addition, SO_2_ can also enhance antioxidative capacity, thereby playing a significant role in alleviating VSR (Figure 2). Hence, the regulatory role of endogenous SO_2_ in VSR would help to further understand the pathogenesis and the potential therapeutic targets of VSR in systemic hypertension and pulmonary hypertension.

However, in the present studies, there are still some limitations. (1) In some studies, investigators used exogenous SO_2_ derivatives to increase the level of SO_2_ in rats or cells. Increasing endogenous SO_2_ content by overexpressing AAT may be more in line with physiology. In addition, the AAT inhibitor HDX used to lower SO_2_ content was not specific. Knocking down AAT to reduce endogenous SO_2_ level together with a rescue experiment is likely a more convincing way to study the significance of endogenous SO_2_. (2) As a gaseous molecule, the mechanisms by which SO_2_ exerts its function need to be further explained. (3) Previous studies showed that gaseous molecules, such as NO, CO, H_2_S, and SO_2_, played an important role in the pathogenesis of VSR [54–57]. However, whether there are any interactions among these gaseous molecules when they function remains to be further studied. (4) Effective treatment for hypertension and pulmonary hypertension is the clinical problem to be solved. Since the regulatory role of VSR in systemic hypertension and pulmonary hypertension is gaining more and more interest, the treatment of VSR has become a new area of the diseases. The regulation of endogenous SO_2_ on VSR would likely become a new therapeutic target. Recently, benzothiazole sulfinate (BTS), a water-soluble SO_2_ donor, was reported to exhibit slow and pH-dependent SO_2_ release ability in aqueous solutions. And it also had SO_2_-like vasorelaxant effect on rat aorta rings [58]. Although the biological activities and validity of BTS still need more exploration, the discovery of potentially better donors would undoubtedly provide a great help and potential for the future clinical application of SO_2_. Meanwhile, although the* in vivo* experiments suggested that the exogenous supplement of SO_2_ derivatives could play a protective role in hypertension and pulmonary hypertension, it still requires more validation in clinical trials to confirm its effects. Furthermore, there are still important things to note: (1) the suitable dosage. Although the current dosage of SO_2_ derivatives (Na_2_SO_3_/NaHSO_3_) used in animal model is 0.54 mmol/kg: 0.18 mmol/kg, we should still take into account the difference between rats and human beings; (2) the potential biomarker. Since the studies suggest that the development of hypertension and pulmonary hypertension is associated with alteration of SO_2_/AAT pathway, it is speculated whether SO_2_ or AAT would become a biomarker for the diseases. Of course, multiple clinical works need to be done to investigate the relationship between endogenous SO_2_/AAT pathway and the severity or survival rate of the diseases.

In summary, SO_2_ inhibited VSMC proliferation, promoted VSMC apoptosis, inhibited the synthesis of extracellular collagen but promoted its degradation, and enhanced antioxidative capacity, thereby playing a significant role in attenuating VSR, which ultimately alleviated systemic hypertension and pulmonary hypertension. Therefore, the understanding of the relationship between endogenous SO_2_ and VSR offers important insight into the pathogenesis of VSR and provides a potential therapeutic target of systemic hypertension and pulmonary hypertension. In the future, with the depth of the study, we are confident to believe that greater progress would be made in the field of SO_2_ biology and medicine to improve the prognosis of disease and enhance the life quality of patients.
